# *Shigella flexneri*-Encoded E3 Ubiquitin Ligase IpaH2 Reveals Plakophilin-2 as a Host Restriction Factor for Sindbis Virus

**DOI:** 10.3390/ijms27114808

**Published:** 2026-05-27

**Authors:** Aaron Embry, Emily A. Rex, David F. Schad, Daniel M. Deng, Neal M. Alto, Don B. Gammon

**Affiliations:** Department of Microbiology, University of Texas Southwestern Medical Center, Dallas, TX 75390, USA

**Keywords:** togavirus, Sindbis virus, *Shigella flexneri*, bacterial effectors, E3 ubiquitin ligase, antiviral immunity

## Abstract

Arthropod-borne viruses (arboviruses) cause a wide range of acute and chronic diseases and represent a growing global health burden. Although some vaccines exist, antiviral therapies remain limited. Identifying host restriction factors may enable new therapeutic strategies. We previously showed that bacterial effector proteins can serve as tools to uncover innate immune defenses. Here, we used a bacterial effector screen in bat cells to identify host factors restricting the arboviruses Sindbis virus (SINV) and O’nyong’nyong virus (ONNV). Several effectors enhanced infection by both viruses. However, we found the *Shigella flexneri*-encoded E3 ubiquitin ligase IpaH2 to selectively promote SINV replication. IpaH2 enhanced SINV infection across multiple mammalian cell lines, suggesting that it targets a conserved antiviral mechanism, and this effect required IpaH2 E3 ubiquitin ligase activity. Screening of putative IpaH2 host targets identified via ubiquitin-activated interaction trap (UBAIT) assays revealed the host factors ATP-binding cassette sub-family F member 3 (ABCF3) and Plakophilin-2 (PKP2) to play roles in restricting SINV infection. While ABCF3 broadly restricted multiple viruses, PKP2 specifically limited SINV replication, indicating a virus-specific restriction factor. These findings demonstrate that bacterial effector screening can identify both broadly acting and virus-specific host defenses, providing insight into antiviral mechanisms and potential therapeutic targets.

## 1. Introduction

Arthropod-borne viruses (arboviruses) comprise a large group of viruses that are transmitted from blood-feeding insects to a wide variety of mammalian hosts, including humans. As a result, they encounter different host immune pathways among the numerous species they infect. Bats are a diverse group of mammals encompassing >1000 species and are found in virtually all global regions populated by humans. Importantly, bats act as natural reservoirs for viruses affecting humans, including certain severe acute respiratory syndrome-related coronaviruses (SARS-CoVs), filoviruses (e.g., Marburg virus), and rhabdoviruses (e.g., rabies virus) [1,2,3,4]. While much attention has focused on bats as reservoirs of the above-mentioned pathogenic viruses, we know relatively little regarding arbovirus–bat host interactions [5]. However, there is growing evidence that bats are also potential reservoirs for arboviruses that can be transmitted by arthropod vectors to humans, such as togaviruses [e.g., Sindbis virus (SINV), Ross River virus (RRV), and O’nyong’nyong virus (ONNV)] [5,6].

As our understanding of the bat immune system is still limited and has largely been gleaned from comparative genomics and transcriptomic profiling of virus-infected bat cells, developing functional assays to identify bat factors relevant to viral restriction is of utmost importance [1,2,3,4,5,6,7,8,9]. We recently used bacterial effectors as tools to identify host antiviral factors in invertebrate and vertebrate cells, and identified effectors that enhance arboviral replication in normally restrictive conditions [10,11]. Bacterial effectors are proteins injected into eukaryotic host cells through needle-like bacterial secretion systems that often function to subvert the host immune response [12,13,14,15,16,17]. Therefore, the identification of bacterial effectors that enhance viral replication can indicate that these effectors target host immune factors that may be broadly antimicrobial/antiviral, and thus can be used as molecular tools to identify relevant host immune machinery.

Here, we show that bacterial effectors can be used as functional tools to identify arbovirus restriction factors encoded by mammalian species, such as bats. By screening a library of 210 bacterial effectors in bat cells, we identified 24 effectors that enhance the replication of either SINV or ONNV in mammalian cells. Notably, we identified the poorly characterized *Shigella flexneri*-encoded E3 ubiquitin ligase IpaH2 as specifically promoting SINV replication. Given that IpaH2 did not affect the replication of other closely related togaviruses, we hypothesized that IpaH2 may target a SINV-specific restriction factor. Prior work by us and others demonstrated that the *S. flexneri* IpaH family of E3 ubiquitin ligases function as immune evasion proteins by targeting specific host innate immunity factors for degradation in the proteasome [10,11,18,19,20,21,22]. For example, we identified the related effector IpaH4 as an E3 ubiquitin ligase capable of enhancing multiple arboviral infections across various host species, and downstream studies led to the identification of mammalian RNF214 proteins as novel, conserved host immunity factors and key targets of IpaH4-mediated ubiquitination and degradation [10,11]. Here, we found the SINV-specific enhancement by IpaH2 to be reproducible in cells derived from a range of mammalian species, including humans, suggesting that IpaH2 also targets a conserved mammalian immunity factor. By screening putative host substrates of IpaH2 identified by ubiquitin-activated interaction trap (UBAIT) assays [20], we found the host proteins, ATP-binding cassette sub-family F member 3 (ABCF3) and plakophilin-2 (PKP2), to both be depleted by IpaH2 expression in cells and to mediate the restriction of SINV in mammalian cells. However, while ABCF3 depletion in cells also promoted the replication of other viruses, PKP2 depletion did not, suggesting that PKP2 is a SINV-specific restriction factor. Together, our work demonstrates that bacterial effector screening can not only provide a functional platform for identifying broad enhancers of viral infections [10,11], but also virus-specific rescue factors conserved across various host species.

## 2. Results

### 2.1. Bacterial Effectors Enhancing SINV and ONNV Infections in R06E Cells

**Figure 1:**
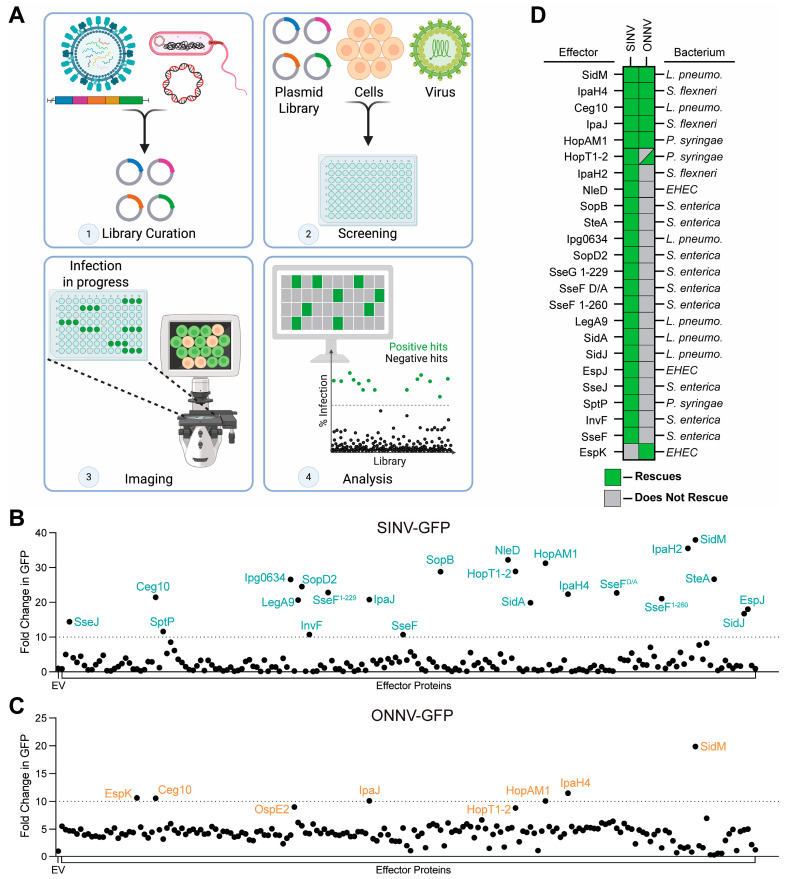
Screening pipeline identifies bacterial effectors enhancing SINV and ONNV infection in R06E bat cells. (**A**) Schematic of screening methodology and pipeline development. (**B**,**C**) Fold change in viral reporter readout in R06E bat cells, normalized to empty vector control treatments for SINV-GFP (MOI = 0.001) (**B**) and ONNV-GFP (MOI = 0.001) (**C**). The cutoff for mean fold change (*N* = 3) in GFP signal was set to ≥10, represented by the dotted horizontal line. (**D**) Summary of bacterial effector proteins that rescued at least one virus. Green blocks mark effectors that rescue the indicated virus. Half-green blocks indicate effectors that failed to reach the threshold in the screen but were validated in downstream confirmatory experiments (Figure 2). The virus is indicated by the column header. The bacterium encoding each effector is noted to the right: *Shigella flexneri* (*S. flexneri*), *Pseudomonas syringae* (*P. syringae*), *Salmonella enterica* (*S. enterica*), *Legionella pneumophila* (*L. pneumophila*), *Enterohemorrhagic Escherichia coli* O157:H7 (EHEC). Additional effector proteins from *Yersinia pseudotuberculosis* and *Bartonella henselae* were also screened but did not rescue arbovirus replication. Created in BioRender. Embry, A. (2026) https://BioRender.com/fy7nppk (accessed on 24 May 2026).

Previously, we have shown that, despite the ability of the arboviruses RRV (a togavirus) and vesicular stomatitis virus (VSV; rhabdovirus) to productively infect mammalian cells, their replication can be further enhanced by expression of specific bacterial effectors that inhibit host immunity factors [11]. This suggested that we could use bacterial effectors as tools to identify novel host immunity factors restricting arbovirus infection in mammalian cells. Thus, we developed and optimized a pipeline for functional screens that identify bacterial effectors encoding immune evasion functions by identifying effectors that can enhance the replication of fluorescent reporter-encoding arboviruses (Figure 1A). Here, we sought to use this pipeline to identify effectors that could enhance the replication of two related togaviruses, SINV and ONNV, in bat-derived R06E cells to complement our prior study (Figure 1A–C) [11].

We examined SINV and ONNV replication in R06E cells, derived from the Egyptian fruit bat (*Rousettus aegyptiacus*), for several reasons: (1) The genome has been sequenced [6]. (2) Our prior work suggested that RRV, another togavirus, could replicate in these cells [11]. (3) Reports by others suggest that *R. aegyptiacus* animals are naturally infected with viruses belonging to the *Togaviridae* family [5]. Additionally, *R. aegyptiacus* is a known reservoir for other pathogenic viruses affecting humans, such as the Marburg virus [1,7,8]. Thus, an improved understanding of *R. aegyptiacus* immunity may have implications for other viral diseases, beyond arboviruses.

Using our lentivirus-based library encoding 210 distinct effector proteins, we transduced R06E cells and expressed effectors for 48 h before viral challenge. These cells were then infected with GFP-reporter strains of SINV (SINV-GFP) or ONNV (ONNV-GFP) for 24 h before imaging, as previously described [10,11]. Effector proteins that enhanced viral GFP signals by >10-fold were considered “hits” in our screens. We identified 24 effectors capable of enhancing viral infections, 23 of which improved SINV infection (Figure 1B) and 6 that promoted ONNV (Figure 1C), with 5 that overlapped between both viruses (Figure 1D).

**Figure 2:**
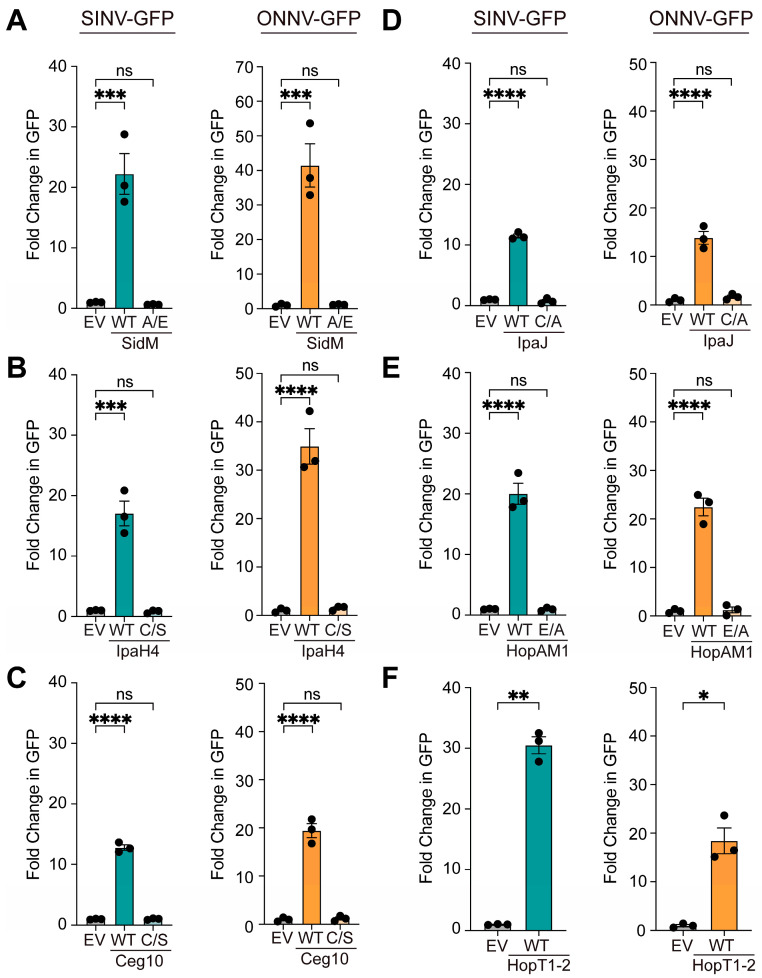
Validation of top hits from the bacterial effector screen in R06E cells. (**A**–**F**) Fold change in GFP-reporter readout for SINV-GFP (MOI = 0.001, Blue) or ONNV-GFP (MOI = 0.001, Orange) after transfection of wild-type (WT) or mutant effector pCDNA3 constructs: (**A**) *L. pneumophila*-encoded SidM. (**B**) *S. flexneri*-encoded IpaH4. (**C**) *L. pneumophila*-encoded Ceg10. (**D**) *S. flexneri*-encoded IpaJ. (**E**) *P. syringae*-encoded HopAM1. (**F**) *P. syringae*-encoded HopT1-2. Quantitative data are means ± SEM; *N* = 3. Statistical significance in graphs was determined with one-way ANOVA and Dunnett’s post-test; ns (not significant), * = *p* < 0.05, ** = *p* < 0.01, *** = *p* < 0.001, **** = *p* < 0.0001.

### 2.2. The Catalytic Activities of Bacterial Effectors Are Required for Enhanced Viral Infection

We next wanted to confirm effector expression and determine if known catalytic or domain activities are required for the top bacterial effector hits we identified. To do this, we cloned the top six effectors enhancing both SINV and ONNV (Figure 1D) into the pCDNA3 mammalian expression vector with a C-terminal Flag-tag, and expressed them in R06E cells for 48 h. Cells were then either (1) challenged with infection by SINV-GFP or ONNV-GFP (Figure 2A–F) or (2) collected for immunoblot to confirm the expression of both the wild-type (WT) or mutant forms of the proteins (Figure S1A–F). Representative microscopy images for R06E cells challenged with either SINV-GFP or ONNV-GFP can be seen in Figure S2. Consistent with our previous findings [11], the WT effectors were all capable of promoting arboviral infections, while the mutant versions of these effectors (SidM^A435E^, IpaH4^C339S^, Ceg10^C159S^, IpaJ^C90A^, or HopAM1^E191A^) lacked these viral phenotypes. Our labs and others have previously described the functions of these effectors and identified catalytic sites responsible for their activities [10,23,24,25]. In brief, SidM is a guanine nucleotide exchange factor [23,24], IpaH4 is an E3 ubiquitin ligase [10,11], Ceg10 is a putative cysteine protease or acetyltransferase [10,26], IpaJ is a protease that cleaves an array of N-myristoylated proteins [27], and HopAM1 is an NAD^+^ hydrolase [28,29]. Of note, HopT1-2 lacks a known catalytic or functional domain, and thus only the WT version of this effector was tested (Figure 2F). It is important to recognize that HopT1-2 was only scored as a hit in the SINV screen (Figure 1B–D) and not in our ONNV screen. However, when we re-tested the HopT1-2-Flag pCDNA3 construct in our validation experiments, we observed a significant increase in ONNV infection (Figure 2F), suggesting that HopT1-2 was a false negative in our large-scale screens. Given these mixed results, we denoted HopT1-2 as a half-green tile in Figure 1D. We suspect the robust expression achieved with plasmid-based expression used in our validation screens may explain why HopT1-2 was not identified as a hit in our original screens using lentivirus-based effector expression. Thus, it is important to note that our screens may have resulted in false negatives if robust effector expression was not achieved by lentivirus transduction.

**Figure 3:**
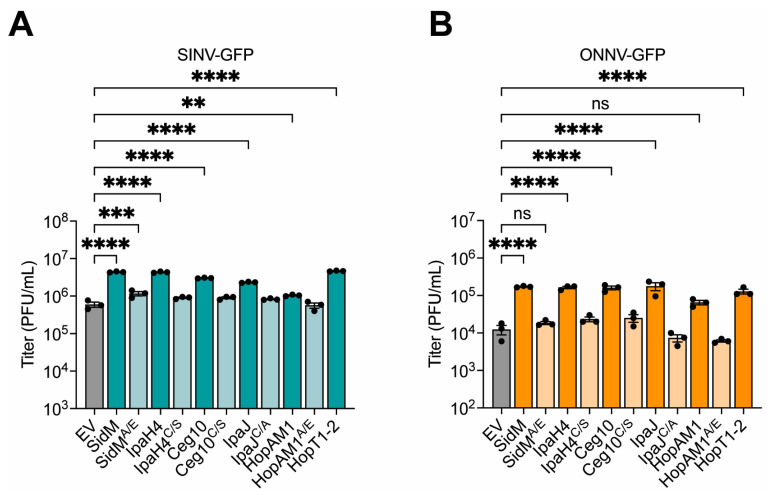
Viral titers output from R06E cells expressing the top 6 bacterial effectors. Titer of supernatants from R06E cell cultures treated as described in Figure 2, infected for 24 h with (**A**) SINV-GFP (MOI = 0.001, Blue) or (**B**) ONNV-GFP (MOI = 0.001, Orange) after transfection of wild-type (WT) or mutant effector pCDNA3 constructs. Quantitative data are means ± SEM; *N* = 3. Statistical significance in graphs was determined with one-way ANOVA and Dunnett’s post-test; ns (not significant), ** = *p* < 0.01, *** = *p* < 0.001, **** = *p* < 0.0001. Comparisons not shown were not statistically significant.

To further validate that the differences we observed in GFP reporters are indeed in response to increased viral production, we next titered supernatants from R06E cells expressing the top six effectors following 24 h of viral challenge with either SINV-GFP (Figure 3A) or ONNV-GFP (Figure 3B). Consistent with our GFP-reporter observations, the WT effectors were all capable of promoting SINV production, while most of the mutants lacked the ability to do so, with SidM^A/E^ as the one exception. This mutant displayed a minor but statistically significant increase in SINV viral titer, but it was markedly reduced compared to wild-type SidM enhancement (Figure 3A). Additionally, SidM^A/E^ failed to enhance ONNV-GFP titers (Figure 3B). Although HopAM1 showed a trend for an increase in ONNV-GFP titer, it failed to reach the statistically significant levels it displayed for SINV-GFP titers. Regardless, we believe these results indicate that, for the effectors with known functions, these proteins require their activities to enhance arboviral infection of mammalian cells.

### 2.3. IpaH2 Selectively Enhances SINV Infection in U2OS Cells

**Figure 4:**
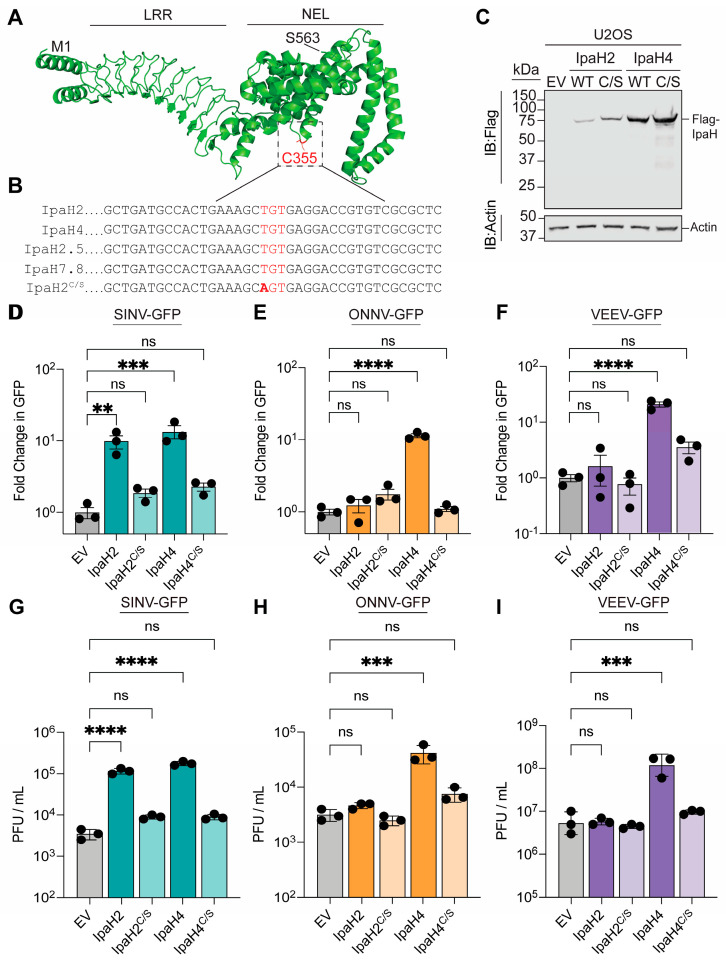
The *S. flexneri*-encoded E3 ubiquitin ligase IpaH2 specifically enhances SINV infection. (**A**) Alphafoldv3 structural predication of IpaH2 with starting methionine (M1) and terminal serine (S563) denoted in black, and catalytic cysteine (C355) denoted in red. N-terminal leucine-rich repeat domain (LRR) and C-terminal novel E3-ligase (NEL) domains labeled above. (**B**) Nucleotide alignment of various IpaH NEL domains highlighting conservation of the catalytic cysteine, and the mutation introduced to generate the inactive serine mutant. (**C**) Representative immunoblot of *N* = 2 for Flag-tagged wild-type (WT) and mutant (C/S) IpaH constructs expressed in U2OS cells. (**D**–**F**) Fold change in reporter readout for U2OS cells expressing IpaH constructs for 24 h prior to viral challenge with SINV-GFP (MOI = 0.001, **D**), ONNV-GFP (MOI = 0.001, **E**), or VEEV-GFP (MOI = 0.0001, **F**). (**G**–**I**) Titer of supernatants from U2OS cell cultures treated as described in (**D**–**F**). Quantitative data in (**G**–**I**) are means ± SEM; *N* = 3. Statistical significance in graphs was determined with one-way ANOVA and Dunnett’s post-test; ns (not significant), ** = *p* < 0.01, *** = *p* < 0.001, **** = *p* < 0.0001.

When analyzing our screen data, we noticed that the uncharacterized IpaH2 effector specifically enhanced SINV but not ONNV replication (Figure 1D). This was in contrast to our prior studies showing that a related family member, IpaH4, is a potent effector capable of enhancing the infections of many different single-stranded RNA (ssRNA) arboviruses by targeting host RNF214 proteins [11]. The IpaH family of proteins is secreted by the intracellular bacterial pathogen *S. flexneri* [30,31,32]. These effectors have two domains, a sequence variable leucine-rich repeat (LRR) domain and a C-terminal novel E3-ligase domain (NEL), which is highly conserved (Figure 4A) [11]. By aligning IpaH2 to other IpaH proteins (including those previously studied by our lab), we identified C355 as the conserved catalytic cysteine residue in IpaH2 (Figure 4B) [10,19,20]. We then substituted this cysteine residue with a serine (C/S), which is known to abolish the E3-ligase activity of other members of this family [10,18,19,33]. Furthermore, we and others have shown that IpaH family members are capable of autoubiquitination, which can promote IpaH protein turnover [10,18,19,33]. This may explain why WT IpaH2 expression is less pronounced than the IpaH2^C/S^ mutant (Figure 4C). To test whether IpaH2 E3 ubiquitin ligase activity was required to enhance SINV infection, and to determine if IpaH2 could enhance SINV infection in other mammalian host species, we expressed the WT or C/S form of IpaH2 in human U2OS cells and then challenged them with SINV-GFP. As a positive control for enhancement of SINV replication, we also expressed the related IpaH4 effector, which we have previously found to broadly enhance the replication of multiple arboviruses in human cells [11]. Immunoblotting confirmed that both the WT and C/S mutant constructs were detectable 48 h post-transfection at the time of viral challenge (Figure 4C). When cells expressing these constructs were challenged with SINV-GFP, both IpaH2 and IpaH4 could enhance the GFP signal > 10-fold, suggesting that these effectors can promote the replication of SINV (Figure 4D). Surprisingly, IpaH2 did not rescue other togaviruses related to SINV such as ONNV-GFP and Venezuelan encephalitis equine virus (VEEV-GFP). In contrast, IpaH4 was capable of enhancing ONNV-GFP and VEEV-GFP signals (Figure 4E,F) and titers (Figure 4G–I). Together, these data demonstrate that IpaH2 is a SINV-specific enhancing factor, and the catalytic activity of IpaH2 is necessary to mediate this enhancement of SINV infection.

### 2.4. IpaH2 Enhances SINV Replication in Cells Derived from Multiple Mammalian Host Species

**Figure 5:**
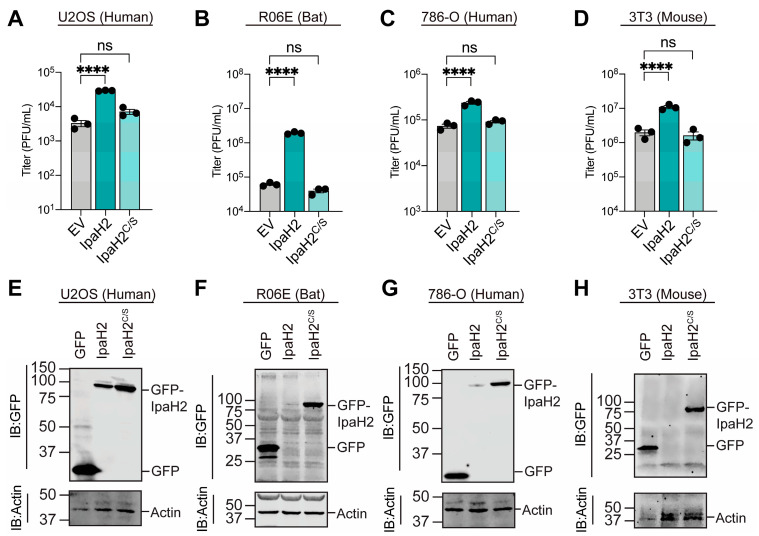
IpaH2 enhances SINV infection in various host species cell lines. (**A**–**D**) Viral titers from supernatants of cells expressing GFP constructs following 24 h of viral challenge with SINV-GFP (MOI = 0.001). Various cell lines include (**A**) U2OS human cells, (**B**) R06E bat cells, (**C**) 786-O human cells, and (**D**) 3T3 mouse cells. (**E**–**H**) Representative immunoblot of *N* = 2 for lysates from the various indicated cell lines after 48 h expressing the denoted C2 construct. Quantitative data in (**A**–**D**) are means ± SEM; *N* = 3. Statistical significance in graphs was determined with one-way ANOVA and Dunnett’s post-test; ns (not significant), **** = *p* < 0.0001.

Given that the host tropism for IpaH2-mediated viral enhancement of SINV infection seemed to remain present in both bat and human cell lines, we next sought to determine if other cell lines and species would retain this phenotype. To test this, we expressed IpaH2 expression vectors in various mammalian cell lines including bat R06E, murine 3T3, and human U2OS or 786-O for 48 h and then challenged these cells with SINV-GFP. We then measured viral titers 24 h post-infection. We found expression of WT IpaH2 to be capable of enhancing SINV infections in all cell lines, while the mutant (C/S) was consistently unable to enhance SINV-GFP infection (Figure 5A–D). It is important to note that this phenotype was observed despite the more robust expression of the mutant compared to WT IpaH2 (Figure 5E–H). These findings suggest that IpaH2 may target broadly conserved antiviral machinery as opposed to cell- or species-specific restriction mechanisms.

### 2.5. Identification of Antiviral Host Proteins ABCF3 and PKP2 as Putative Targets of IpaH2

**Figure 6:**
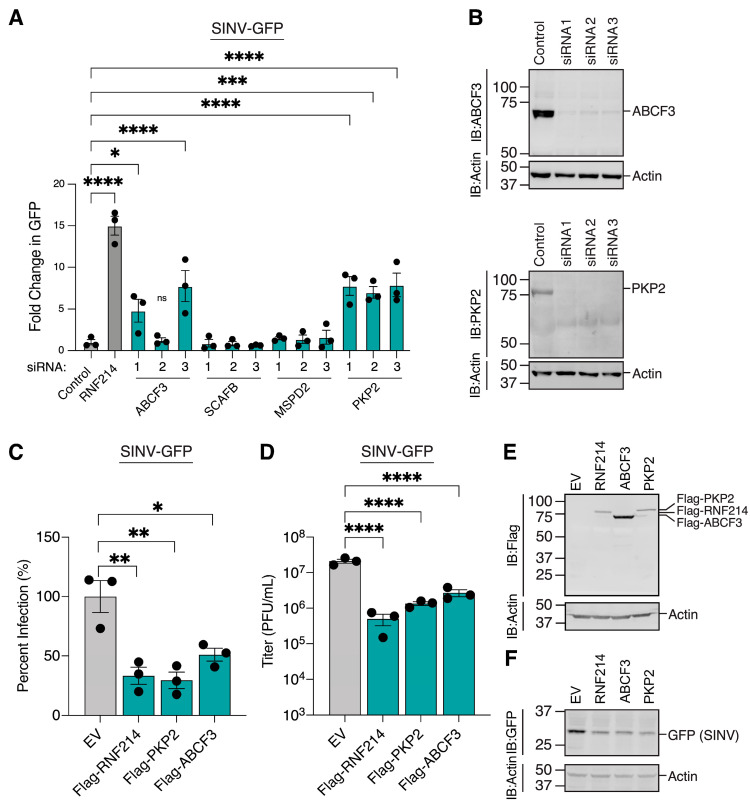
Identification of putative IpaH2 substrates that affect SINV infection. (**A**) Fold change in GFP signal from SINV-GFP 24 h post-infection (MOI = 0.001) in U2OS cells after siRNA knockdown of designated host proteins. GFP signals were normalized to the scramble control-treated cells and include RNF214 knockdown as a positive control. (**B**) Representative immunoblot of *N* = 2 for endogenous ABCF3 (Top) or PKP2 (Bottom) levels following siRNA knockdown in U2OS cells for 48 h. (**C**) Normalized GFP signal from SINV-GFP 24 h post-infection (MOI = 0.1) in U2OS cells overexpressing the designated host gene for 48 h. (**D**) Viral titers from supernatants of cells from panel (**C**). (**E**) Representative immunoblot of overexpression of pcDNA3 expression plasmids from (**C**,**D**). (**F**) Representative immunoblot of SINV-encoded GFP following infections from (**C**,**D**). All immunoblots are representative of *N* = 2. Quantitative data in A are means ± SEM; *N* = 3. Statistical significance in graphs was determined with one-way ANOVA and Dunnett’s post-test; ns (not significant), * = *p* < 0.05, ** = *p* < 0.01, *** = *p* < 0.001, **** = *p* < 0.0001. Comparisons not shown were not statistically significant.

Although the host targets of IpaH2 are still largely unknown, a prior study used UBAIT assays in human cell lysates to identify putative host targets of various IpaH family members, including IpaH2 [20]. Using these data, we used RNA interference (RNAi) to screen the putative human targets of IpaH2: ATP-binding cassette sub-family F member 3 (ABCF3), SR-Related CTD-Associated Factor 11 (SCAFB), Motile Sperm Domain Containing 2 (MSPD2), and Plakophilin-2 (PKP2) for antiviral phenotypes. Additionally, we knocked down human RNF214 (a target of IpaH4) as a positive control for increased virus replication, given our prior identification of this antiviral factor (Figure 6A) [11]. We used three independent siRNAs to knock down each of these targets individually and then challenged these U2OS cells with SINV-GFP infection. Of these host targets, only ABCF3 and PKP2 depletion resulted in statistically significant increases in SINV-GFP replication in at least two of the three siRNA treatments (Figure 6A). Importantly, we used commercial antibodies to immunoblot lysates treated as in Figure 6A to confirm knockdown of ABCF3 and PKP2. Based on densitometric analysis, we observed ~90% or more reduction in these target proteins by their respective siRNAs (Figure 6B).

Given that the IpaH2 phenotype is SINV-specific, we next sought to determine if depletion of host ABCF3 or PKP2 impacted the replication of additional arboviruses, including VEEV-GFP and VSV-GFP. While ABCF3 knockdown had no impact on VEEV-GFP infections, its depletion enhanced VSV-GFP infection (Figure S3A,B), suggesting that ABCF3 may restrict certain togaviruses (SINV) and rhabdoviruses (VSV) but could not explain the SINV-specific enhancement we observe after IpaH2 expression. Conversely, PKP2 knockdown did not enhance VEEV-GFP nor VSV-GFP infections (Figure S3A,B), suggesting that it may be a SINV-specific restriction factor, possibly explaining why IpaH2 promotes SINV replication but not VEEV replication (Figure 4D–F).

To further assess the impact of these host proteins on viral replication, we next tested whether overexpression of these proteins would reduce viral infection. We cloned both ABCF3 and PKP2 into pcDNA3 with Flag-tags and expressed them in U2OS cells for 48 h prior to viral challenge with SINV-GFP (Figure 6C,D). As a positive control, we included Flag-RNF214, a host protein we have shown to suppress viral infection upon overexpression [11]. Consistent with our knockdown data (Figure 6A and Figure S3A,B), overexpression of Flag-ABCF3 or Flag-PKP2 was sufficient to suppress viral GFP corresponding to SINV-GFP infection (Figure 6C). Furthermore, viral titers of the supernatants of these infections were determined via plaque assay. Consistent with the GFP data, overexpression of these Flag-tagged host proteins resulted in a ~10–15-fold reduction in SINV-GFP titers (Figure 6D). We confirmed expression of all constructs via immunoblot (Figure 6E), as well as blotted to confirm the lower levels of GFP visualized via immunofluorescence (Figure 6F) when Flag-tagged RNF214, ABCF3, or PKP2 were overexpressed. These data suggest that, while ABCF3 can affect the replication of multiple RNA viruses, PKP2 seems to display an SINV-specific antiviral phenotype. Phylogenetic analyses of PKP2 suggest it is highly conserved across vertebrate species (Figure S3C), with bat PKP2 being >89% identical to human and >78% identical to mouse PKP2 (Figure S3D). This high degree of conservation is consistent with our observation that IpaH2 enhances SINV infection across various mammalian hosts (Figure 4) and suggests that PKP2 may have a conserved role in host defense across multiple host species.

**Figure 7:**
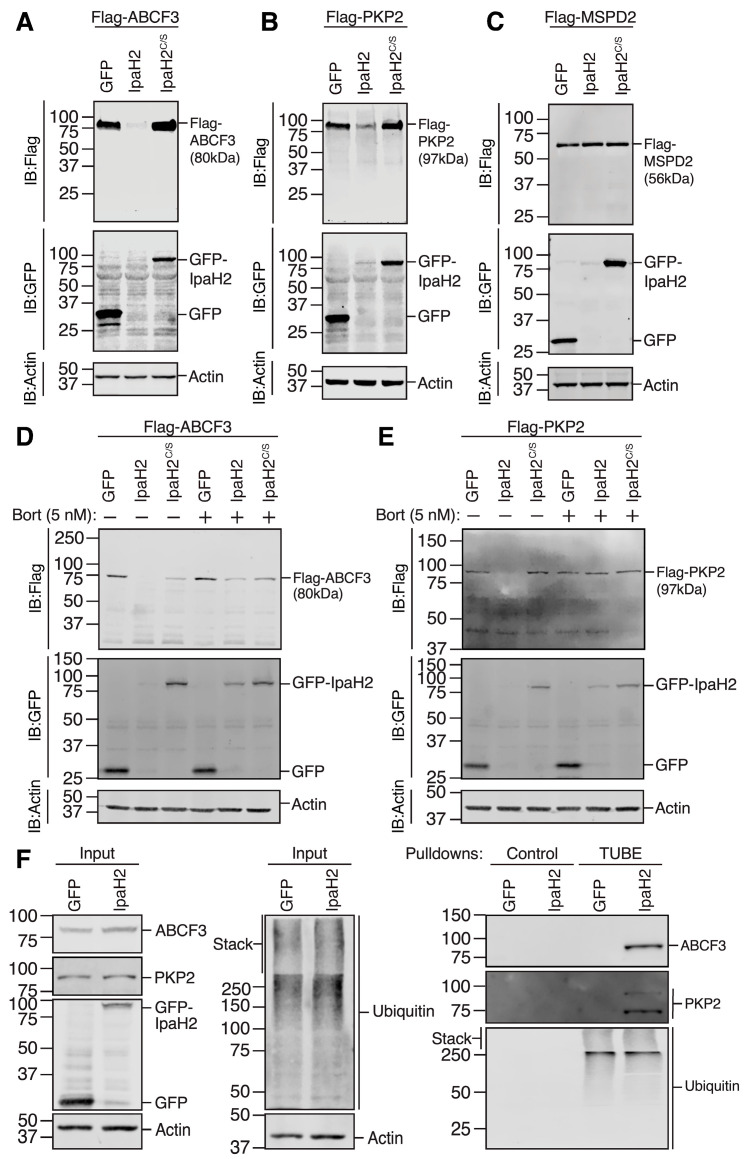
IpaH2 promotes ABCF3 and PKP2 depletion and ubiquitination in cells. (**A**–**C**) Representative immunoblot of co-expression assay using Flag-tagged (**A**) ABCF3, (**B**) PKP2, or (**C**) MSPD2 co-transfected into HEK293T cells with GFP-IpaH2 or GFP-IpaH2^C/S^ catalytic mutant expression vectors. (**D**,**E**) Representative immunoblot of co-expression assay of (**D**) Flag-ABCF3 or (**E**) Flag-PKP2 in the presence of Bortezomib (Bort) added 24 h post-transfection for 8 h. (**F**) Representative immunoblot of TUBE (or control TUBE) pulldowns of ubiquitinated protein fractions from U2OS cells expressing GFP (control) or GFP-IpaH2 for 24 h. Bort was added to cultures 6 h post-transfection for the duration of the experiment. All immunoblots are representative of *N* = 2.

To further investigate if ABCF3 or PKP2 may be substrates of IpaH2, we utilized the Flag-tagged ABCF3 and PKP2 plasmid and co-expressed them in HEK293T cells along with GFP-tagged IpaH2 to assess their expression. When ABCF3 or PKP2 were co-expressed with either GFP (control) or the mutant GFP-IpaH2^C/S^, both ABCF3 and PKP2 constructs were expressed to similar levels (Figure 7A,B). However, in the presence of WT GFP-IpaH2, both ABCF3 and PKP2 levels were depleted, suggesting that they may be targeted by IpaH2. In contrast, expression of a Flag-tagged version of MSPD2 (which did not produce a viral phenotype in our siRNA knockdown experiments (Figure 6A)) showed no difference in expression regardless of the co-expressed IpaH2 construct (Figure 7C). This suggests that the altered expression levels for ABCF3 and PKP2 are likely not due to IpaH2 non-specifically suppressing protein expression from any vector, but rather that these proteins may be specifically targeted by IpaH2 in a manner dependent upon IpaH2 E3 ligase activity. Given that the IpaH family of proteins tends to ubiquitinate substrates for proteasomal degradation, we next wanted to determine if inhibition of the proteasome could rescue the expression of these constructs. We repeated the co-expression experiments in the presence of Bortezomib (Bort), a potent inhibitor of proteasomal activity, and again probed for the Flag-tagged proteins. We observed that, upon Bort treatment, levels of both Flag-ABCF3 and Flag-PKP2 were enhanced compared to control (no Bort) conditions (Figure 7D,E). These data suggest that IpaH2-mediated reduction in ABCF3 and PKP2 is proteasome-dependent. It should be noted that in some cases, we had difficulty detecting WT IpaH2 under control conditions (e.g., Figure 7E) but were able to detect WT IpaH2 in the presence of Bort. This is consistent with our aforementioned speculation that, like other IpaH proteins, IpaH2 likely undergoes autoubiquitination and rapid turnover by the proteasome.

Next, we wanted to confirm if IpaH2 promotes the ubiquitination of ABCF3 and PKP2. Thus, we used tandem ubiquitin-binding entities (TUBEs) to pull down all ubiquitinated host proteins from U2OS cell lysate after transfection of constructs expressing either a GFP control protein or GFP-IpaH2. Immunoblot analysis of the TUBE pulldown fractions with anti-ubiquitin antibodies demonstrated a clear and specific enrichment for ubiquitinated proteins compared to control TUBE pulldowns. Importantly, both ABCF3 and PKP2 were only detectable in the TUBE pulldowns (i.e., ubiquitinated protein fractions) in the presence of GFP-IpaH2 (Figure 7F). These data indicate that the expression of IpaH2 promotes the ubiquitination of PKP2 and ABCF3. Given that IpaH family members function as E3 ubiquitin ligases, these findings suggest that IpaH2 targets these host factors for ubiquitin modification, leading to their proteasomal degradation. Collectively, our data suggest that ABCF3 and PKP2 may be targets of IpaH2 and that they can play antiviral roles against either unrelated viruses (ABCF3) or against SINV specifically (PKP2).

## 3. Discussion

Here, we have utilized bacterial effector proteins as tools to understand the restriction of arboviruses in mammalian cells. By screening a library of 210 effectors encoded by seven distinct bacterial pathogens, we identified 24 effectors capable of enhancing ONNV and/or SINV infection of bat cells. Bats are natural reservoirs for many viruses, with increasing evidence that they may also harbor togaviruses [2,5,9]. Thus, bat cells may be relevant hosts to identify eukaryotic host defenses that restrict togavirus replication. Our work suggests that bat cell-encoded togavirus restriction mechanisms can be overcome by the expression of specific effector proteins, indicating that bacterial effectors could be useful tools for identifying and characterizing innate immune defenses in these hosts. Moreover, identifying virus restriction and rescue phenotypes in bat cells can lead to the discovery of conserved components of antiviral immunity found in other mammalian species, such as humans, highlighting the evolutionary conservation of these host pathways.

In previous studies, our screens have focused on bacterial effectors that broadly rescue diverse virus infections in various host species [10,11,34]. However, in this study, we sought to investigate mechanisms underlying effectors that rescue viral replication in a virus-specific manner. Through our bacterial effector screens, we uncovered several effectors that specifically enhance SINV or ONNV infection (Figure 1). In this study, we focused on the *Shigella flexneri*-encoded effector IpaH2, given that it is an uncharacterized member of bacterial E3 ubiquitin ligases and because other IpaH members often encode immune evasion functions [10,11,19,20]. We found that IpaH2 catalytic activity was required to enhance SINV-GFP infections, and this phenotype remained consistent across a variety of host species, including bat, human, and mouse cells. Together, this suggested that IpaH2 targets host immunity machinery broadly conserved across different host species.

By screening putative host targets of IpaH2 for antiviral phenotypes, we identified ABCF3 and PKP2 as mediators of virus restriction. Interestingly, ABCF3 has been shown to display antiviral activity against flaviviruses, such as West Nile virus (WNV), through interactions with the interferon-inducible immunity factor OAS1B [35,36]. In this context, ABCF3 is proposed to form a complex with OAS1B that promotes flavivirus restriction. Our observations now suggest that ABCF3 knockdown enhances SINV and VSV infection (Figure 6A and Figure S3B), indicating that this host protein may function as a broadly acting antiviral factor capable of restricting RNA viruses from multiple families rather than functioning in a virus-specific manner [36]. Although it is unclear how ABCF3 may restrict viral infection, a prior study implicated ABCF3 proteins in translation regulation [37]. It is possible that IpaH2-mediated antagonism of ABCF3 may dampen host translation, which could, in turn, block host cells from mounting effective immune responses. If so, this could help explain how ABCF3 depletion may be beneficial to the replication of both viruses and *S. flexneri*.

The second host protein, PKP2, belongs to a family of scaffolding and signaling molecules involved in diverse cellular processes, including cell-to-cell adhesion, cytoskeletal organization, and signal transduction [38]. Among the PKP family members (PKP1-3, with PKP4 more variably defined), PKP2 is particularly notable for its antiviral activity. PKP2 has been shown to restrict influenza A virus through direct interaction with the PB1 subunit of the viral RNA-dependent RNA polymerase (RdRP), thereby inhibiting viral replication [39]. While this provides a clear mechanistic basis for antiviral restriction in the context of orthomyxoviruses, we believe such a model is unlikely to fully explain the SINV-specific phenotype we have observed here. For one, given the conserved nature of togavirus RdRps, one might expect IpaH2 to broadly rescue multiple togaviruses, but we found IpaH2 to only enhance SINV infection and not ONNV or VEEV (Figure 4). However, we cannot rule out the possibility that PKP2 does target togavirus replication machinery, but that certain togaviruses encode antagonists capable of counteracting PKP2 function that are lacking or non-functional in SINV.

We hypothesize that PKP2 restricts SINV through distinct, RdRP-independent mechanisms for several reasons. For one, the identification of PKP2 as a target of the *S. flexneri* effector IpaH2 suggests that PKP2 functions as a general immunity factor targeting both viruses and bacteria. Moreover, *S. flexneri* does not encode an RdRP. Instead, IpaH2-mediated ubiquitination and suppression of PKP2 may disrupt host processes such as junctional integrity [40,41,42] or immune signaling pathways [38,43,44] that are detrimental to bacterial infection. In this light, PKP2 may contribute to barrier function or coordinate signaling pathways that enhance host defenses, and PKP2 inactivation by IpaH2 could facilitate pathogen invasion, dissemination, or immune evasion. It is therefore plausible that togaviruses, such as SINV, may also benefit from suppressing PKP2-dependent signaling to enhance cell-to-cell spread or evade host defenses. Intriguingly, other PKP family members (e.g., PKP4) have emerged from screens as putative proviral factors that promote SINV entry [45], suggesting that the PKP family may play multifaceted roles in virus–host interactions. Future mechanistic studies will be required to define how PKP2 and ABCF3 contribute to antiviral defense, their relative contributions to virus restriction, and whether these mechanisms also play roles in the defense against intracellularly replicating bacterial pathogens, such as *S. flexneri*.

### Limitations of the Study

While we observed a reduction in both ABCF3 and PKP2 of ~90% in our siRNA experiments in Figure 6, we did not have antibodies available to measure the knockdown efficiency of the other putative IpaH2 substrates (e.g., SCAFB, MSPD2). Thus, if knockdown of these other putative IpaH2 targets was inefficient, we may have missed detecting antiviral phenotypes with these other factors. Although ABCF3 and PKP2 were detected in IpaH2 UBAIT assays, which often detect direct E3 ubiquitin ligase substrates, and our data demonstrate that IpaH2 promotes ubiquitination and proteasome-dependent depletion of ABCF3 and PKP2, we have not shown that IpaH2 directly ubiquitinates these host substrates in vitro. Thus, although unlikely, it is possible that IpaH2 targets other host factors and that this targeting indirectly leads to the depletion of ABCF3 and PKP2.

## 4. Materials and Methods

### 4.1. Cell Lines and Cell Culture

Mammalian cell lines were maintained at 37 °C in a 5% CO_2_ atmosphere. U2OS cells were cultured in DMEM supplemented with 10% FBS containing 1% non-essential amino acids (NEAAs), 1% L-glutamine, and 1% antibiotic/antimycotic (ThermoFisher, Waltham, MA, USA**)**. R06E cells were cultured in DMEM:F12 supplemented with 10% FBS and 1% antibiotic/antimycotic (ThermoFisher, Waltham, MA, USA). BSC-40 cells were cultured in MEM supplemented with 5% FBS containing 1% non-essential amino acids (NEAAs), 1% L-glutamine, and 1% antibiotic/antimycotic (ThermoFisher, Waltham, MA, USA) [11].

### 4.2. Viruses

Stock preparation, culture of recombinant viruses, and titration by fluorescent foci/plaque assay on BSC-40 cells was performed as previously described [10,11]. Viral inocula were incubated with cells for 1 h in serum-free media before the addition of complete media for the remainder of the infection. Where indicated, complete media containing ActD (ThermoFisher, Waltham, MA, USA) at the indicated dose was added for the remainder of the infection.

### 4.3. Plasmid Constructs for Mammalian Cell Expression

The 210 bacterial effector protein coding sequences were generated in pENTR/D as previously described [11]. Briefly, PCR-amplified bacterial genomic DNA sequences were cloned into pENTR/D-TOPO (ThermoFisher, Waltham, MA, USA) using topoisomerase I. Source organisms were *Pseudomonas syringe* pv. Tomato (ATCC BAA-871D-5), *Legionella pneumophila* Philadelphia-1 (ATCC 33152D-5), *Salmonella typhimurium* LT2 (gift of Jack Dixon, University of California San Diego), EHEC H7:O157 (gift of Vanessa Sperandio, University of Wisconsin-Madison), *S. flexneri* M90T (gift of Jack Dixon, University of California San Diego), and *Bartonella henselae* Houston-1 (gift of Alexei Savchenko, University of Toronto). For expression in mammalian cells, the library was cloned into the lentiviral expression vector pTRIP-CMV-IVSb-IRES-TagRFP using Gateway LR Clonase II (ThermoFisher, Waltham, MA, USA).

N-terminal Flag human ABCF3, PKP2, and MSPD2 were generated via gateway cloning into pcDNA3 as previously described for other constructs [11].

### 4.4. Lentivirus Production

HEK293T cells were plated in poly-D-lysine-coated 6-well plates at 400,000 cells/well in 2 mL/well to yield a confluency of 50% the following day. Media was changed to 1.5 mL/well DMEM containing 3% FBS and 1% NEAA. Cells were then transfected with 200 ng/well pCMV-VSVG, 800 ng/well pCMV-Gag/Pol, and 1000 ng/well pTRIP-CMV-Effector-IVSb-IRES-TagRFP using X-tremeGENE 9 (Sigma Aldrich, Burlington, MA, USA; Table S2) and OptiMEM (ThermoFisher, Waltham, MA, USA; Table S2). Following 6 h of incubation, media was changed to 1.5 mL/well DMEM with 3% FBS and 1% NEAA. At 48 h post-media change, the supernatant was collected and replaced with fresh media. After an additional 24 h, the supernatant was again collected. Supernatants from both time points were pooled, centrifuged at 3000× *g* for 5 min to remove cell debris, and combined with HEPES to a concentration of 20 mM and polybrene to a concentration of 4 µg/mL. Lentivirus stocks were then arrayed in v-bottom 96-well plates and stored at −80 °C.

### 4.5. Bacterial Effector Screens and Fluorescence Microscopy

Cells were seeded into 96-well clear-bottom dishes, transduced with the lentivirus effector library in quadruplicate for 48 h, and then challenged with indicated GFP-reporter viruses. At the indicated times post-infection, cells were stained as previously described with CellTracker Dye, fixed with PFA, and imaged by the UT Southwestern Medical Center High-Throughput Screening Core using an INCell Analyzer 6000 (Molecular Devices, San Jose, CA, USA) scope equipped with 405, 488, and 561 nm lasers using a 10× objective. Four images were taken per well so that fluorescence signals per well resulted from an average of these four images. Signals across all 4 replicate wells for each effector treatment were then used to determine GFP-reporter virus infection levels. Image analysis was conducted using Fiji 2.14.0 (National Institutes of Health, Bethesda, MD, USA) to quantify the percent area of each field of view containing GFP signals and these signals were normalized to the CellTracker Dye signal to account for cell numbers. Finally, normalized GFP signals for each effector treatment were plotted as a fold change in the GFP signal relative to cells transduced with lentivirus-targeted luciferase (negative control). Validation assays to confirm virus-enhancing phenotypes after transfection of pcDNA3.1 expression plasmids encoding Flag-tagged effector constructs were conducted and analyzed in a similar manner, except an empty pcDNA3.1 vector was used as a negative control treatment.

### 4.6. General Transfection Protocols

Unless otherwise stated, 100,000 cells were plated into 24-well dishes, transfected with 500 ng of expression vectors for 48 h prior to manipulation (e.g., protein extraction or virus infection). For U2OS cell expression of the pcDNA3.1 constructs, 500 ng of vector was mixed with 100 μL of OptiMEM media and 1.5 μL of Lipofectamine 2000 Reagent (ThermoFisher, Waltham, MA, USA). This mixture was incubated at room temperature for 20 min, and media on the cells was changed to 500 μL OptiMEM; then the transfection mixture was added dropwise to wells, incubated overnight, and media was then changed to complete DMEM ~16 h post-transfection.

For R06E transfection experiments, as well as HEK293T transfection, 500 ng of vector was mixed with 50 μL of Opti-MEM and 1 μL of FuGENE HD Transfection Reagent (Promega, Madison, WI, USA). This mixture was incubated at room temperature for 20 min before being added dropwise to the well. Cells were then incubated for 48 h prior to further manipulation or infection.

Transient siRNA-mediated knockdown was achieved by reverse transfection of U2OS cells with 8 pmol siRNA and 1.5 μL of Lipofectamine 2000 according to the manufacturer’s protocol. Cells were transfected for 48 h and then subjected to GFP-reporter virus infection to assess replication phenotypes.

### 4.7. Co-Transfection Assays

In total, ~75,000 cells were co-transfected with 150 ng of target pcDNA3.1 vectors encoding full-length Flag-tagged targets and 350 ng of pEGFP-C2 encoding either GFP, GFP-IpaH2, or GFP-IpaH2C/S using 1.5 μL of X-tremeGENE 9 and 50 μL of OptiMEM (Sigma Aldrich, Burlington, MA, USA). After 24 h, cells were harvested directly in 1X Laemmli buffer containing β-mercaptoethanol. Protein extracts were then subjected to SDS-PAGE and subsequent immunoblotting with the indicated antibodies.

### 4.8. Immunoblotting

Immunoblotting was conducted as previously described with commercial primary antibodies and secondary antibodies conjugated with infrared dyes and a Li-Cor Odyssey scanner [10,11].

### 4.9. Statistical Analyses

Graphs were presented as mean values ± SEM with individual data points shown. At least three independent experiments were conducted for all quantitative experiments shown, where statistical analyses were applied. All statistical analyses were performed with Prism software v10.0.2 (GraphPad, San Diego, CA, USA), and statistical tests used are indicated in respective figure legends. Statistical significance (*p* < 0.05) between compared groups is indicated in figures as either ns (not significant), * = *p* < 0.05, ** = *p* < 0.01, *** = *p* < 0.001, or **** = *p* < 0.0001.

### 4.10. Key Reagents and Resources

See Table S1 for information regarding key reagents and resources used in this study.

## Data Availability

All relevant data are within the manuscript, supporting information, or cited where appropriate. Inquiries may be directed to the corresponding author.

## Supplementary Materials

The following supporting information can be downloaded at: https://www.mdpi.com/article/10.3390/ijms27114808/s1.

## Abbreviations

The following abbreviations are used in this manuscript:

SINV	Sindbis virus
ONNV	O’nyong’nyong virus
VSV	Vesicular stomatitis virus
IpaH	Invasion plasmid antigen H
ABCF3	ATP-binding cassette sub-family F member 3
PKP2	Plakophilin-2
RRV	Ross River virus
VEEV	Venezuelan equine encephalitis virus
GFP	Green fluorescent protein
*R. aegyptiacus*	*Rousettus aegyptiacus*
*S. flexneri*	*Shigella flexneri*
*P. syringae*	*Pseudomonas syringae*
*S. enterica*	*Salmonella enterica*
*L. pneumophila*	*Legionella pneumophila*
EHEC	Enterohemorrhagic *Escherichia coli* O157:H7
SEM	Standard error of mean
ANOVA	Analysis of Variance
LRR	Leucine-rich repeat domain
NEL	Novel E3-ligase domain
UBAIT	Ubiquitin-activated interaction trap
SCAFB	SR-related CTD-associated factor 11
MSPD2	Motile sperm domain containing 2
siRNA	Small interfering RNA
OAS1B	2′-5′ Oligoadenylate synthetase 1B
DMEM	Dulbecco’s Modified Eagle Medium
FBS	Fetal bovine serum
NEAA	Non-essential amino acids
PFA	Paraformaldehyde
SDS-PAGE	Sodium dodecyl sulfate polyacrylamide gel electrophoresis
Bort	Bortezomib

## References

[1] Amman B.R., Bird B.H., Bakarr I.A., Bangura J., Schuh A.J., Johnny J., Sealy T.K., Conteh I., Koroma A.H., Foday I. (2020). Isolation of Angola-like Marburg virus from Egyptian rousette bats from West Africa. Nat. Commun..

[2] Hoffmann M., Müller M.A., Drexler J.F., Glende J., Erdt M., Gützkow T., Losemann C., Binger T., Deng H., Schwegmann-Weßels C. (2013). Differential sensitivity of bat cells to infection by enveloped RNA viruses: Coronaviruses, paramyxoviruses, filoviruses, and influenza viruses. PLoS ONE.

[3] Letko M., Seifert S.N., Olival K.J., Plowright R.K., Munster V.J. (2020). Bat-borne virus diversity, spillover and emergence. Nat. Rev. Microbiol..

[4] Makenov M.T., Boumbaly S., Tolno F.R., Sacko N., N’fAtoma L.T., Mansare O., Kolie B., Stukolova O.A., Morozkin E.S., Kholodilov I.S. (2023). Marburg virus in Egyptian Rousettus bats in Guinea: Investigation of Marburg virus outbreak origin in 2021. PLoS Neglected Trop. Dis..

[5] Fagre A.C., Kading R.C. (2019). Can Bats Serve as Reservoirs for Arboviruses?. Viruses.

[6] PPavlovich S.S., Lovett S.P., Koroleva G., Guito J.C., Arnold C.E., Nagle E.R., Kulcsar K., Lee A., Thibaud-Nissen F., Hume A.J. (2018). The Egyptian Rousette Genome Reveals Unexpected Features of Bat Antiviral Immunity. Cell.

[7] Amman B.R., Koroma A.H., Schuh A.J., Conteh I., Sealy T.K., Foday I., Johnny J., Bakarr I.A., Whitmer S.L.M., Wright E.A. (2024). Sosuga Virus Detected in Egyptian Rousette Bats (*Rousettus aegyptiacus*) in Sierra Leone. Viruses.

[8] Balkema-Buschmann A., Rissmann M., Kley N., Ulrich R., Eiden M., Groschup M.H. (2018). Productive Propagation of Rift Valley Fever Phlebovirus Vaccine Strain MP-12 in Rousettus aegyptiacus Fruit Bats. Viruses.

[9] Kading R.C., Borland E.M., Mossel E.C., Nakayiki T., Nalikka B., Ledermann J.P., Crabtree M.B., Panella N.A., Nyakarahuka L., Gilbert A.T. (2022). Exposure of Egyptian Rousette Bats (*Rousettus aegyptiacus*) and a Little Free-Tailed Bat (Chaerephon pumilus) to Alphaviruses in Uganda. Diseases.

[10] Embry A., Baggett N.S., Heisler D.B., White A., de Jong M.F., Kocsis B.L., Tomchick D.R., Alto N.M., Gammon D.B. (2024). Exploiting bacterial effector proteins to uncover evolutionarily conserved antiviral host machinery. PLoS Pathog..

[11] Embry A., Schad D.F., Rex E.A., Alto N.M., Gammon D.B. (2025). Bacterial effector screening reveals RNF214 as a virus restriction factor in mammals. PLoS Pathog..

[12] Alto N.M., Orth K. (2012). Subversion of cell signaling by pathogens. Cold Spring Harb. Perspect. Biol..

[13] Costa T.R.D., Harb L., Khara P., Zeng L., Hu B., Christie P.J. (2021). Type IV secretion systems: Advances in structure, function, and activation. Mol. Microbiol..

[14] Deng W., Marshall N.C., Rowland J.L., McCoy J.M., Worrall L.J., Santos A.S., Strynadka N.C.J., Finlay B.B. (2017). Assembly, structure, function and regulation of type III secretion systems. Nat. Rev. Microbiol..

[15] Galán J.E. (2009). Common themes in the design and function of bacterial effectors. Cell Host Microbe.

[16] Ham H., Sreelatha A., Orth K. (2011). Manipulation of host membranes by bacterial effectors. Nat. Rev. Microbiol..

[17] Hayes C.S., Aoki S.K., Low D.A. (2010). Bacterial contact-dependent delivery systems. Annu. Rev. Genet..

[18] Ashida H., Kim M., Schmidt-Supprian M., Ma A., Ogawa M., Sasakawa C. (2010). A bacterial E3 ubiquitin ligase IpaH9.8 targets NEMO/IKKgamma to dampen the host NF-kappaB-mediated inflammatory response. Nat. Cell Biol..

[19] de Jong M.F., Liu Z., Chen D., Alto N.M. (2016). Shigella flexneri suppresses NF-κB activation by inhibiting linear ubiquitin chain ligation. Nat. Microbiol..

[20] Hansen J.M., de Jong M.F., Wu Q., Zhang L.-S., Heisler D.B., Alto L.T., Alto N.M. (2021). Pathogenic ubiquitination of GSDMB inhibits NK cell bactericidal functions. Cell.

[21] Li P., Jiang W., Yu Q., Liu W., Zhou P., Li J., Xu J., Xu B., Wang F., Shao F. (2017). Ubiquitination and degradation of GBPs by a Shigella effector to suppress host defence. Nature.

[22] Luchetti G., Roncaioli J.L., Chavez R.A., Schubert A.F., Kofoed E.M., Reja R., Cheung T.K., Liang Y., Webster J.D., Lehoux I. (2021). Shigella ubiquitin ligase IpaH7.8 targets gasdermin D for degradation to prevent pyroptosis and enable infection. Cell Host Microbe.

[23] Brombacher E., Urwyler S., Ragaz C., Weber S.S., Kami K., Overduin M., Hilbi H. (2009). Rab1 guanine nucleotide exchange factor SidM is a major phosphatidylinositol 4-phosphate-binding effector protein of Legionella pneumophila. J. Biol. Chem..

[24] Zhu Y., Hu L., Zhou Y., Yao Q., Liu L., Shao F. (2010). Structural mechanism of host Rab1 activation by the bifunctional Legionella type IV effector SidM/DrrA. Proc. Natl. Acad. Sci. USA.

[25] Li Q., Xu L., Yin C., Liu Z., Li Y., Yuan Y., Hu Y., Jiao X. (2020). The Invasion Plasmid Antigen J (IpaJ) from Salmonella Inhibits NF-κB Activation by Suppressing IκBα Ubiquitination. Infect. Immun..

[26] Gao J., Xu W., Zhou Z., Lu L., Ge Z., Wang W., Lu Y., Ge H. (2026). Legionella effector Ceg10 acetylates RPS20 to inhibit host translation and induce cell cycle arrest. Proc. Natl. Acad. Sci. USA.

[27] Burnaevskiy N., Fox T.G., Plymire D.A., Ertelt J.M., Weigele B.A., Selyunin A.S., Way S.S., Patrie S.M., Alto N.M. (2013). Proteolytic elimination of N-myristoyl modifications by the Shigella virulence factor IpaJ. Nature.

[28] Eastman S., Smith T., Zaydman M.A., Kim P., Martinez S., Damaraju N., DiAntonio A., Milbrandt J., Clemente T.E., Alfano J.R. (2022). A phytobacterial TIR domain effector manipulates NAD+ to promote virulence. New Phytol..

[29] Manik M.K., Shi Y., Li S., Zaydman M.A., Damaraju N., Eastman S., Smith T.G., Gu W., Masic V., Mosaiab T. (2022). Cyclic ADP ribose isomers: Production, chemical structures, and immune signaling. Science.

[30] Ashida H., Sasakawa C. (2015). Shigella IpaH Family Effectors as a Versatile Model for Studying Pathogenic Bacteria. Front. Cell. Infect. Microbiol..

[31] Ashida H., Toyotome T., Nagai T., Sasakawa C. (2007). Shigella chromosomal IpaH proteins are secreted via the type III secretion system and act as effectors. Mol. Microbiol..

[32] Singer A.U., Rohde J.R., Lam R., Skarina T., Kagan O., DiLeo R., Chirgadze N.Y., Cuff M.E., Joachimiak A., Tyers M. (2008). Structure of the Shigella T3SS effector IpaH defines a new class of E3 ubiquitin ligases. Nat. Struct. Mol. Biol..

[33] Keszei A.F.A., Sicheri F. (2017). Mechanism of catalysis, E2 recognition, and autoinhibition for the IpaH family of bacterial E3 ubiquitin ligases. Proc. Natl. Acad. Sci. USA.

[34] Embry A., Gammon D.B. (2024). Abortive Infection of Animal Cells: What Goes Wrong. Annu. Rev. Virol..

[35] Govande A.A., Babnis A.W., Urban C., Habjan M., Hartmann R., Kranzusch P.J., Pichlmair A. (2023). RNase L-activating 2’-5’ oligoadenylates bind ABCF1, ABCF3 and Decr-1. J. Gen. Virol..

[36] Peterson E., Shippee E., Brinton M.A., Kaur P. (2019). Biochemical characterization of the mouse ABCF3 protein, a partner of the flavivirus-resistance protein OAS1B. J. Biol. Chem..

[37] Hirose T., Horvitz H.R. (2014). The translational regulators GCN-1 and ABCF-3 act together to promote apoptosis in *C. elegans*. PLoS Genet..

[38] Bass-Zubek A.E., Godsel L.M., Delmar M., Green K.J. (2009). Plakophilins: Multifunctional scaffolds for adhesion and signaling. Curr. Opin. Cell Biol..

[39] Wang L., Fu B., Li W., Patil G., Liu L., Dorf M.E., Li S. (2017). Comparative influenza protein interactomes identify the role of plakophilin 2 in virus restriction. Nat. Commun..

[40] Godsel L.M., Dubash A.D., Bass-Zubek A.E., Amargo E.V., Klessner J.L., Hobbs R.P., Chen X., Green K.J. (2010). Plakophilin 2 couples actomyosin remodeling to desmosomal plaque assembly via RhoA. Mol. Biol. Cell..

[41] Nagler S., Ghoreishi Y., Waschke J., Schlegel N. (2020). Plakophilins 2 and 3 are involved in desmosome formation in the intestine. FASEB J..

[42] Nagler S., Ghoreishi Y., Kollmann C., Kelm M., Gerull B., Waschke J., Burkard N., Schlegel N. (2023). Plakophilin 2 regulates intestinal barrier function by modulating protein kinase C activity in vitro. Tissue Barriers.

[43] Pérez-Hernández M., Marrón-Liñares G.M., Schlamp F., Heguy A., van Opbergen C.J.M., Mezzano V., Zhang M., Liang F.-X., Cerrone M., Delmar M. (2020). Transcriptomic Coupling of PKP2 With Inflammatory and Immune Pathways Endogenous to Adult Cardiac Myocytes. Front. Physiol..

[44] Cerrone M., Montnach J., Lin X., Zhao Y.-T., Zhang M., Agullo-Pascual E., Leo-Macias A., Alvarado F.J., Dolgalev I., Karathanos T.V. (2017). Plakophilin-2 is required for transcription of genes that control calcium cycling and cardiac rhythm. Nat. Commun..

[45] Alvarez P.A., Tang A., Winters D.M., Kaushal P., Medina A., Nieto M.V., Kaczor-Urbanowicz K.E., Amant F.S., Reyes B.R., Kaake R.M. (2025). Old World alphaviruses use distinct mechanisms to infect brain microvascular endothelial cells for neuroinvasion. Cell Rep..

