# Supplementary figures and images for: Shigella flexneri-Encoded E3 Ubiquitin Ligase IpaH2 Reveals Plakophilin-2 as a Host Restriction Factor for Sindbis Virus
Source: Int J Mol Sci. 2026 May 27;27(11):4808. doi: 10.3390/ijms27114808 (PMC13256859; doi:10.3390/ijms27114808)

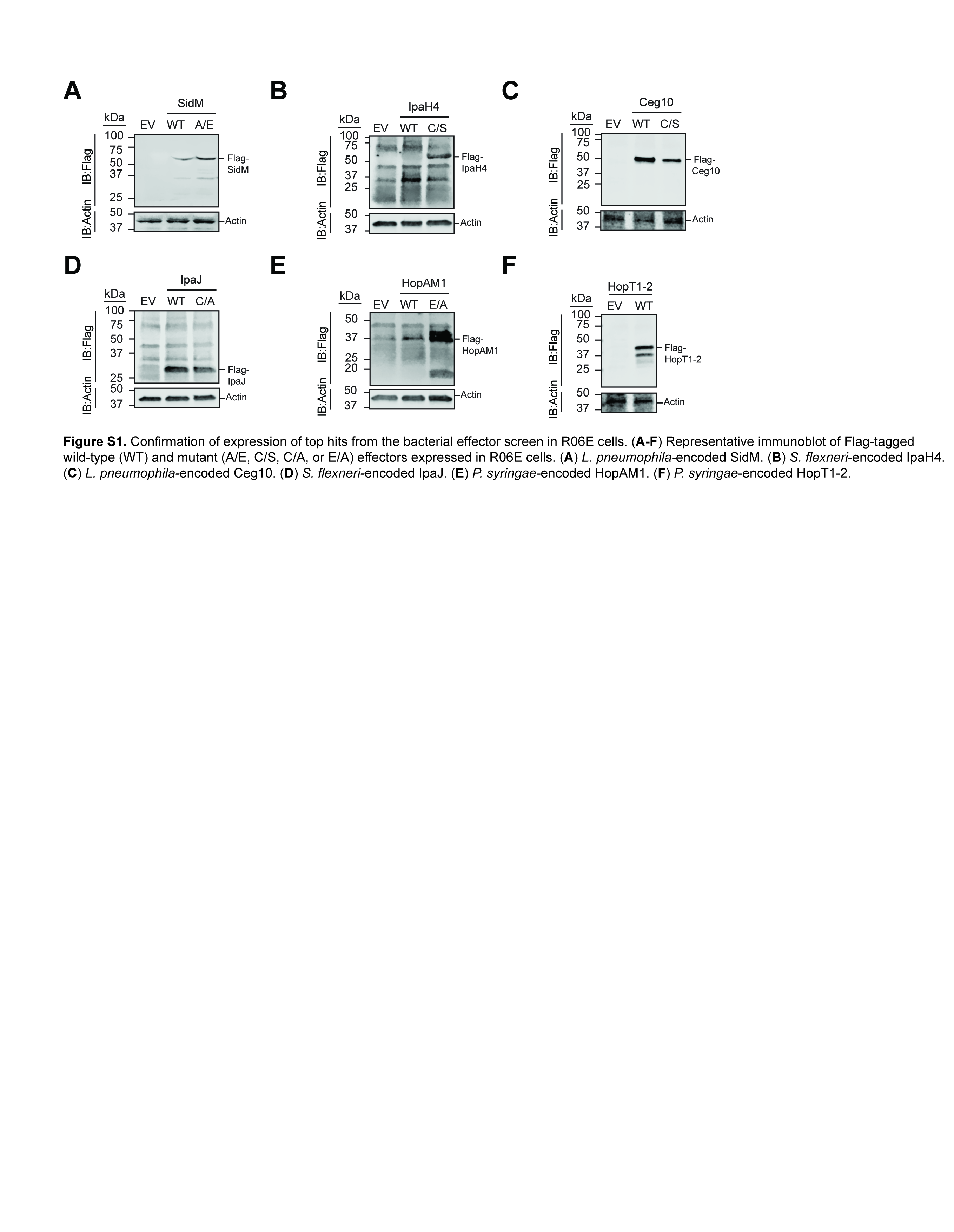

Supplement: Supplementary file 1 [file ijms-27-04808-s001.zip › FigureS1.tif]

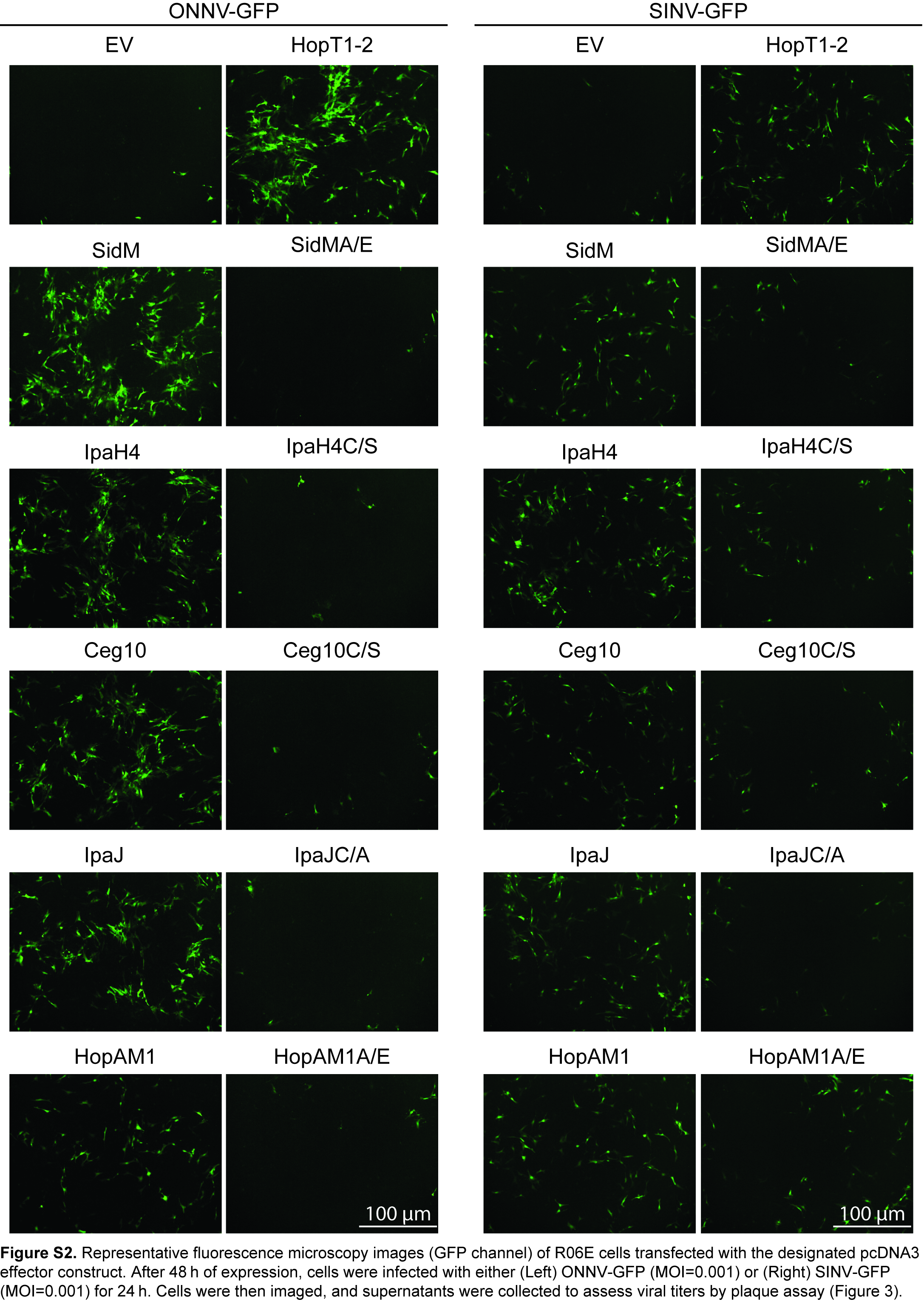

Supplement: Supplementary file 1 [file ijms-27-04808-s001.zip › FigureS2.tif]

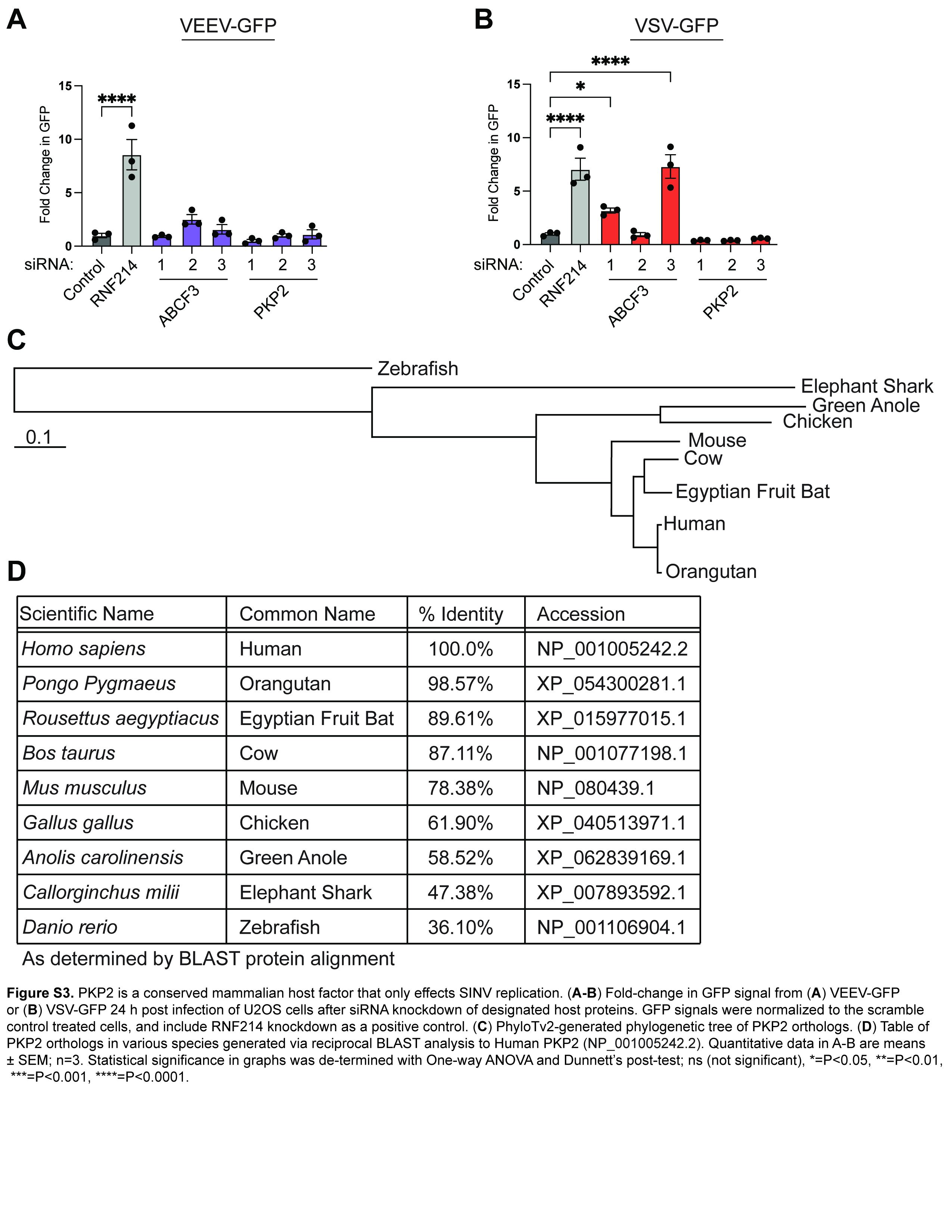

Supplement: Supplementary file 1 [file ijms-27-04808-s001.zip › FigureS3.tif]
